# Commentary: Mild endoplasmic reticulum stress ameliorates lpopolysaccharide-induced neuroinflammation and cognitive impairment via regulation of microglial polarization

**DOI:** 10.3389/fnagi.2018.00192

**Published:** 2018-06-25

**Authors:** Sachchida N. Rai, Walia Zahra, Hareram Birla, Saumitra S. Singh, Surya P. Singh

**Affiliations:** Department of Biochemistry, Institute of Science, Banaras Hindu University, Varanasi, India

**Keywords:** endoplasmic reticulum, lipopolysaccharide, neuroinflammation, cognitive impairment, microglia

In this article, Wang and colleagues have discussed the non-harmful levels of Endoplasmic reticulum (ER) stress in rats focusing mainly on primary microglia. Specifically, they sought to investigate the regulation of lipopolysaccharide (LPS) driven neuroinflammation in male Sprague-Dawley rats through mild ER-stress (MERS). In experiment 1, to determine the extent of unfolded protein response (UPR), they measured expression of phosphorylated total protein kinase RNA-like ER kinase (p-PERK), phosphorylated eukaryotic translation initiation factor 2α (p-EIF2α), phosphorylated inositol-requiring protein 1α (p-IRE1α), spliced X-box-binding protein-1 (XBP1s), XBP1u, activating transcription factor-4 (ATF4) and CCAAT/enhancer-binding protein homologous protein (CHOP) through western blot and Immunofluorescence (Wang et al., 2017). During ER stress, IRE1α gets phosphorylated & activated and cuts unspliced XBP1u mRNA into spliced XBP1s mRNA which further encodes XBP1 protein (Gardner et al., 2013). They found that expressions of p-IRE1α and XBP1s were considerably increased on administration of different doses of tunicamycin (TM), while the expression of XBP1u was significantly reduced. Expression of hippocampal p-PERK, p-EIF2α, ATF4, and CHOP were also assessed. p-PERK causes phosphorylation of EIF2α (Walter and Ron, 2011; Hetz et al., 2015) which on prolonged phosphorylation induces paradoxical translation of ATF4 mRNA into its corresponding protein, in turn inducing upregulation of pro-apoptotic components such as CHOP (Gardner et al., 2013).

Furthermore, Caspase-3 and cleaved caspase-3 expressions were assessed in the CA1 region of the hippocampus. Increased expression of p-PERK and p-EIF2-α were seen at a range of different doses of TM administration, but ATF4, CHOP and cleaved caspase-3 were only elevated at the highest dose of TM. Thus, the authors concluded that low doses of TM, i.e.; 0.3 and 3 μg/2 μl *in vivo* & 0.5 and 5 ng/ml *in vitro* led to modest UPR without cell or organism lethality as assessed by TUNEL labeling, while the higher concentrations of 30 μg/2 μl *in vivo* and 50 ng/ml *in vitro* have shown serious ER perturbations and a robust UPR. In experiment 2, the authors studied the role of MERS in LPS-induced neuroinflammation and cognitive impairment in rats. MERS was induced by using 3 μg/2 μl TM and treated with or without sodium 4-phenylbutyrate (a stabilizing agent), an hour before the LPS administration both *in vivo* and *in vitro*. This low dose of TM (3 μg) significantly improved freezing behavior and learning trials, indicating its role in protection against memory dysfunction caused by LPS.

They have also shown that TM prevented neurons from undergoing LPS-induced apoptosis. To clarify whether MERS was responsible for neuroprotective activity of TM, they administered rats with 100 mg/kg 4-Phenylbutyric acid (4-PBA) known to reduce ER stress, which at this dose does not affect normal functioning of nervous system. Treatment with 4-PBA significantly reduced expression levels of p-IRE1α and XBP1s as compared to non-PBA treated groups. Also, neuroprotection conferred by TM was partially blocked by concomitant administration of 4-PBA as revealed by increased numbers of TUNEL-positive cells. Therefore, significant reductions in cognitive function in the TM+LPS+4-PBA group confirmed that low dose of TM protects against LPS-induced cognitive dysfunction by inducing MERS which inhibits caspase-3 activation (Wang et al., 2017). As in neurodegenerative disorders, microglia activation indicates an early sign of neuronal death, thus the authors tried to explore the effect of MERS on microglia *in vitro* by measuring expression of microglial genes associated with classic (M1), alternative repair and regeneration (M2a) and, immunomodulation (M2b) (Tang and Le, 2016). The relative expression of classical M1 genes CD86, CD32 and inducible nitric oxide synthase (iNOS), M2a genes YM1/2 and CD206 and, M2b gene suppressor of cytokine signaling 3 were assessed. LPS was shown to significantly increase mRNA levels of M1 and M2b markers in hippocampus compared with levels in the naïve group, while M2a genes were significantly reduced as compared to the naïve group. Alternatively, TM pretreatment led to alteration in balance of M1 and M2 microglia expression patterns in hippocampus significantly increasing expression of M2a genes and decreasing expression of M1 and M2b genes. Thus, the authors concluded that LPS-induced neuroinflammation was significantly attenuated by MERS, leading to a shift of the microglia population from M1/2b to M2a in hippocampus. Further, the authors also used double immunofluorescent staining of iNOS and CD206 with microglial marker Iba1 in the hippocampal CA1 region, to show that TM inhibited LPS-induced microglia activation and shifted the phenotype of microglia toward M2a.

The authors also detected levels of TNF-α, IL-1β, and IL-6 proinflammatory factors thought to play major role in neuroinflammation. They have seen increased expression of these proinflammatory factors induced by LPS, while TM has significantly reduced this inflammatory response. But, the expression levels of these factors were significantly enhanced after 4-PBA co-treatment through reversal of anti-neuroinflammatory effects of TM. Results were further validated *in vitro* by demonstrating that dose of 5 ng/ml of TM was able to induce MERS in microglial culture. They have confirmed that TM has inhibited cytokine production and induced microglial polarization from M1/2b to M2a. Moreover, 4-PBA led to impairment of anti-inflammatory effects and M2a differentiation conferred by TM.

Since authors talk about neuroinflammation, they must have studied role of MERS on Astrogliosis. NF-kB plays important role in neuroinflammation, hence must have been investigated. The biochemical parameters such as Catalase and Lipid Peroxidation must have been checked in the hippocampal region. Moreover, on Page: 8, in line “LPS significantly increased the mRNA levels of M1 and M2a markers in the hippocampus compared with the levels observed in the naïve group”; there should be M2b instead of M2a.

Thus, MERS has an important role in neuroinflammation and cognitive impairment. Since, ER stress has also been seen in astrocytes, an important cell type that plays a vital role in neurodegenerative disorders through neuroinflammation, the question arises whether MERS in astrocyte in LPS-induced neuroinflammation could also have contributed to the beneficial properties of MERS.

Finally, they reported that MERS preconditioning can alleviate neuroinflammation and cognitive impairment induced by LPS, thereby suggesting that moderate level of ER stress can act as a new therapeutic possibility to suspend or delay progression of neurodegenerative diseases (Wang et al., 2017).

## Author contributions

All authors listed have made a substantial, direct and intellectual contribution to the work, and approved it for publication.

### Conflict of interest statement

The authors declare that the research was conducted in the absence of any commercial or financial relationships that could be construed as a potential conflict of interest.
